# Emergency physician mental health during the subacute phase of the COVID-19 pandemic

**DOI:** 10.1017/cem.2020.442

**Published:** 2020-07-03

**Authors:** Rodrick Lim, Huma Ali, Rachel Gagnier, Michelle Marlborough, Sandra Northcott

**Affiliations:** 1 Division of Pediatric Emergency Medicine, Departments of Pediatrics and Medicine, Schulich School of Medicine and Dentistry, London, ON, and the Children's Health Research Institute, London Health Sciences Centre, London, ON; 2 Department of Emergency Medicine, Cumming School of Medicine, Calgary, ON; 3 Department of Psychiatry, Schulich School of Medicine and Dentistry, London, ON

Dear Editor,

The coronavirus disease (COVID-19) pandemic presents unique mental stressors for emergency physicians (EPs). In light of the literature on the mental health of EPs during the severe acute respiratory syndrome pandemic, the well-being of EPs during this prolonged COVID-19 pandemic is of major concern, as they are at significant risk of depression, anxiety, and other mental health complaints.^1–4^

EPs pre-pandemic experienced chronic stress, perceived lack of control, inefficiency, and moral distress, which were all leading to burnout.^5^ Tools that worked well to cope during the acute phase may not work well in the subacute phase, as natural decompression occurs and EPs face the unpredictability and chronicity of COVID-19. We propose that three important themes during the subacute phase are important to identify to support EP mental health: uncertainty, lack of control, and discord.

Uncertainty presents as the ever-evolving clinical presentation of COVID-19, the lack of clarity regarding management of future influenza seasons, insecure personal protective equipment supplies, decentralized conflicting sources of information, continued fear of dying or bringing home illness to our families, and the moral injury and potential legal ramifications of providing suboptimal care.

The lack of control that EPs experience with decisions, such as use of personal protective equipment and procedural protocols, and the instinct to help those in need are tempered by the time-consuming requirements to properly don personal protective equipment. We feel powerlessness and frustration when witnessing the actions of community members disregarding physical distancing and masking guidelines.

When an acute phase ends, the sense of unity dissipates as well. Discord has led to an increased feeling of isolation and lack of support from others and the creation of a more hostile work environment.

To support EPs’ mental health during the prolonged COVID-19 pandemic, we propose the following action items:

1. Clear plan for ongoing physician safety and personal protective equipment
2. Clear centralized approach to COVID-19 assessment and treatment
3. Clear plan for COVID-19 and addressing bed block
4. Re-engage frontline EPs in decisions
5. Loud advocacy group for physicians and their health
6. Regular check-ins (e.g., buddy system, group texts)
7. Respect limits and remove barriers to physician self-care
8. Physicians taking a break away from work before the second wave
9. Provide resources for outside work stressors
10. Team approach: share and celebrate success stories

Through these action items, it is critical that we act now to minimize the pandemic repercussions to physician mental health.
